# 4262 Latinas and Cervical Cancer: A Nursing-Community Collaborative Project for Improving Health in Vulnerable Populations

**DOI:** 10.1017/cts.2020.216

**Published:** 2020-07-29

**Authors:** Maria Elena Ruiz, Efrain Talamantes

**Affiliations:** 1 David Geffen School of Medicine at UCLA; 2 AltaMed Health Services

## Abstract

OBJECTIVES/GOALS: We present findings of an academic-community health agency study that explored knowledge of cervical cancer and risks among Latinas. The collaboration between the UCLA School of Nursing and AltaMed, a community-based health organization provided diverse clinical training and opportunities to decrease disparities in marginalized communities. METHODS/STUDY POPULATION: We developed a 19-item open-ended survey guide (English/Spanish) to explore knowledge, beliefs and practices related to cervical cancer. Eight nursing students (females and males) completed a 10-week public health focused practicum at four clinical sites. Students interviewed volunteer Latinas (N = 51) and recorded their responses. Prior to surveying Latina clients, the nursing instructor developed a script and mentored the student through the recruitment process. The survey included items on the Papanicolaou exam (pap smear), the HPV, beliefs and knowledge of risks for cervical cancer and recommendations for health service delivery. RESULTS/ANTICIPATED RESULTS: The Latina participants ranged in age from 20-50s, 70% spoke English, most were US born (52%) and 29% were from Mexico. The majority had received a Pap exam (88%), but fewer understood the purpose for the Pap (72%) or the association between HPV and cervical cancer (6%). Five major themes emerged: (1) knowledge deficits regarding women’s preventive care, and the HPV vaccine; (2) limited Spanish language educational materials; (3) importance of respectful client-provider interactions; (4) modesty; and 5) scheduling appointments and the importance of a diverse workforce that understand cultural and language nuances. Recommendations included ways to improve health literacy, cervical cancer knowledge, and delivery of culturally specific health care. DISCUSSION/SIGNIFICANCE OF IMPACT: Finding highlight the importance of putting “personalismo” into practice; linking health behaviors, vaccines, and health care to addresses cervical cancer risks. The collaboration maximized student experiences with opportunities build evidence based sustainable programs for vulnerable communities.

